# Microcystin-LR and Cylindrospermopsin Induced Alterations in Chromatin Organization of Plant Cells

**DOI:** 10.3390/md11103689

**Published:** 2013-09-30

**Authors:** Csaba Máthé, Márta M-Hamvas, Gábor Vasas

**Affiliations:** Department of Botany, Faculty of Science and Technology, University of Debrecen, Debrecen H-4010, Egyetem tér 1, Hungary; E-Mails: mathe.csaba@science.unideb.hu (C.M.); hamvas.marta@science.unideb.hu (M.M.-H.); vasas.gabor@science.unideb.hu (G.V.); Tel.: +36-52-512-900; Fax: +36-52-512-943.

**Keywords:** microcystin, cylindrospermopsin, cyanobacterial toxin, plant chromatin, microtubules

## Abstract

Cyanobacteria produce metabolites with diverse bioactivities, structures and pharmacological properties. The effects of microcystins (MCYs), a family of peptide type protein-phosphatase inhibitors and cylindrospermopsin (CYN), an alkaloid type of protein synthesis blocker will be discussed in this review. We are focusing mainly on cyanotoxin-induced changes of chromatin organization and their possible cellular mechanisms. The particularities of plant cells explain the importance of such studies. Preprophase bands (PPBs) are premitotic cytoskeletal structures important in the determination of plant cell division plane. Phragmoplasts are cytoskeletal structures involved in plant cytokinesis. Both cyanotoxins induce the formation of multipolar spindles and disrupted phragmoplasts, leading to abnormal sister chromatid segregation during mitosis. Thus, MCY and CYN are probably inducing alterations of chromosome number. MCY induces programmed cell death: chromatin condensation, nucleus fragmentation, necrosis, alterations of nuclease and protease enzyme activities and patterns. The above effects may be related to elevated reactive oxygen species (ROS) and/or disfunctioning of microtubule associated proteins. Specific effects: MCY-LR induces histone H3 hyperphosphorylation leading to incomplete chromatid segregation and the formation of micronuclei. CYN induces the formation of split or double PPB directly related to protein synthesis inhibition. Cyanotoxins are powerful tools in the study of plant cell organization.

## 1. Introduction

The marine and freshwater habitats are considered to be a source of potential drugs. To date approximately 16,000 natural products have been isolated from marine, freshwater organisms so it is not surprising that these organisms are a wonderful source of biologically active natural products with diverse chemistry and pharmacology too [1]. For example marine organisms produce some of the most cytotoxic compounds ever discovered, but the yields of these compounds are invariably so small that natural sources are unlikely to provide enough material for drug development studies [2].

Marine biotoxins have drawn worldwide attention because of their involvement in human intoxication and the socio-economic impacts brought by those incidents. To understand the chemistry and the mechanism of action of the toxins is important because it helps in developing the adequate countermeasures, such as detection, determination and therapeutic methods. In addition many of the toxins have been found to be useful tools for probing biological or pharmacological phenomena, like use of okadaic acid in protein phosphatase studies [3].

The unicellular life forms such as several algae and cyanobacteria, which can become abundant under favorable environmental conditions in waters, are readily collectable, culturable, and thus, have become good targets of many natural products researchers [4]. In freshwaters the most prominent unicellular organisms are the cyanobacteria which can multiply and can develop huge biomass called as blooms [5]. These prokaryotic organisms not only elaborate the common primary metabolites and pharmaceutically useful compounds but also produce toxic substances. The cyanobacterial toxins are a heterogeneous bioactive group with diverse chemistry [6].

Cyanobacterial toxins are classified as hepatotoxins (microcystins and nodularins), neurotoxins (anatoxins and paralytic shellfish toxins), cytotoxins (cylindrospermopsins) and dermotoxins [5]. Their production can be found in a diverse range of blue-green species. The most studied cyanobacterial toxins are the peptide type microcystins (MCYs) and most recently the alkaloid cylindrospermopsin (CYN). In addition there are also the two cyanotoxin groups, from which human injury has been clearly identified [7]. Although there are many results and cytogenetic studies relating the effects of these cyanobacterial metabolites especially in animal and human cells, for understanding the whole mechanism or applying as tools in pharmacology, it is important to know the effects in different organisms (animal, plant, fungi) at different levels (histology, cytology, molecular biology). Thus, cytogenetic studies may help the understanding of cyanobacterial toxicity in natural habitats.

We are focusing in this review on the two main cyanotoxin-MCY and CYN induced changes of chromatin organization and the possible cellular mechanisms of those alterations in plant cells.

## 2. Cyanotoxins-Microcystins and Cylindrospermopsins

### 2.1. Microcystins

MCYs are cyclic heptapeptides consisting of seven amino acids, including d-amino acids and two unusual amino acids namely, *N*-methyldehydroalanine (Mdha) and a hydrophobic d-amino acid, 3-amino-9-methoxy-2-6,8-trimethyl-10-phenyldeca-4,6-dienoic acid (Adda) [8]. More than 60 structural variants are described, with variation occurring mainly at the two l-amino acids along with alterations to side chains [6]. The hepatotoxic microcystins (MCY) are known to be produced by several bloom forming cyanobacterial genera including *Microcystis*,* Anabaena*, *Planktothrix*,* Nostoc* [5]. The toxic mechanism of MC is the inhibition of protein phosphatases of type 1 and 2A (PP1 and PP2A, [9]). Cysteine-273 of the catalytic subunit of PP-1 (Cysteine-266 of PP2A) binds covalently to the carbonyl group of Mdha of MCY but this is not a requirement for the inhibition [10,11]. It is the introduction of Adda into the hydrophobic groove at the catalytic site of protein phosphatase that renders it inactive [12]. The Adda part of the molecules seems crucial for the toxicity, the loss of Adda or synthetic MCY without Adda are consequently non-toxic [13]. Microcystins containing the naturally occurring Z enantiomer (geometrical isomer) of Adda or linearized MCY are less potent protein phosphatase inhibitors [14]. The IC_50_ of protein phosphatase inhibition by MCY-LR is (0.1–0.25 nM)* in vitro*. Liver damage is one of the most common phenomena in the intoxication of microcystins in animal organisms which starts with loss of membrane integrity, cytoskeletal disorganization, cellular disruption, lipid peroxidation, DNA damage, apoptosis, necrosis, and ultimately death by hemorrhagic shock [15].

### 2.2. Cylindrospermopsins

CYN is a low molecular weight (MW.415) tricyclic alkaloid with guanidino group linked at C7 to hydroxymethyl uracil. Because of the negatively charged sulfate group and the positively charged guanidino group, the molecule is a zwitterion and very soluble in polar solvents [16]. CYN was detected in eight different cyanobacterial species and several producers considered as invasive organisms [7,17]. There are two possible natural epimers at the hydroxyl bridge with same toxicity, cylindrospermopsin and 7-epicylindrospermopsin. CYN in *Umezakia natans* from Japan and in *Aphanizomenon ovalisporum* from Israel samples have the same epimeric form, which is of opposite orientation to the toxin of *C. raciborskii* from Australia [18]. The uracil group of the molecules seems crucial for its mechanism, because without this part the cylindrospermopsin molecule effectively lacks toxicity [16]. The mechanisms of cylindrospermopsin toxicity are under investigation. Uptake of the toxin is relatively rapid since complete and irreversible block of protein synthesis occurs after a 1 h exposure* in vitro*. Inhibition of protein synthesis occurs at the ribosome during the peptide chain elongation step [19]. Evaluating the human and animal exposures, the toxin causes damage to liver, kidneys, lungs, heart, stomach, adrenal glands, the vascular system, and the lymphatic system [20]. Recent data from primary hepatocytes show two routes of toxic action, a rapid route probably through toxicity of a cytochrome P450 (CYP450) oxidation product of the toxin [19]. Glutathione synthesis is also reduced via a CYP450-dependent mechanism [21]. The main genotoxic outcome appears to be DNA fragmentation [22,23], although loss of whole chromosomes has also been shown to occur [22]. 

## 3. Particularities of Plant Chromatin and Microtubule Organization

Plant cells have particularities not found in other eukaryotic cells. These include plastids, the presence of cellulose cell wall, storage vacuoles and plasmodesmata, the latter connecting neighboring cells in tissues (see [24] for an example). Not taking into account the lack of unequivocal evidence for the presence of intermediate filaments in plant cell nucleus, the basic structural features of plant chromatin are similar to all eukaryotic cells. Cytoskeletal elements are relevant to this review, since at least mitotic microtubules (MTs) and microfilaments (MFs) have a crucial role in chromatin organization and dynamics. The particularities of plant cytoskeleton are [25,26,27,28,29]: (i) The presence of preprophase bands (PPBs) at G2 phase and early mitosis containing MT and MF arrays and localized at the future site of cell division; (ii) The presence of endoplasmic microtubules (EMTs) playing a role in regulating PPB formation and stabilizing cell plate formation during cytokinesis; (iii) The presence of phragmoplasts at the end of cell division, that contain MTs and MFs as well and direct vesicle traffic to the site of new cell wall formation at the equatorial plane of dividing cells; (iv) The absence of centrioles and the presence of diffuse microtubule organization centre (MTOC) like structures that make MTs more dynamic than in animal cells. The nucleation of MTs is likely to happen in the cortical cytoplasm and nuclear envelope. Spindle formation starts from multiple MTOCs from both poles of dividing cells. The principles of chromatid/chromosome dynamics during meiosis and mitosis in plant cells are similar to the other eukaryotic cell types. However, they have several particularities in this respect-dyneins as cytoskeleton associated motor proteins involved in chromosome movement are absent here, meaning that chromatid/chromosome regulation machinery is not the same as for animal cells; (v) The preferential arrangement of MTs at the periphery of non-dividing (interphase or differentiated) cells, just beneath the plasma membrane (cortical MTs, CMTs). CMTs play an essential role in the regulation of cell elongation and shape and are probably involved in the positioning of nucleus at the onset of mitosis; (vi) The primary role of actin cytoskeleton in the delivery of organelles such as peroxisomes or mitochondria.

The organization of cytoskeletal elements is strongly dependent on protein phosphorylation/dephosphorylation and on the synthesis of certain regulatory proteins like microtubule associated proteins (MAPs) [30,31,32,33]. For the above reason, due to the relevant particularities of plant cells—with special emphasis on PPB, spindle and phragmoplast formation—it is worthwhile to review the effects of PP1 and PP2A inhibitory MCY and protein synthesis inhibitory CYN in those systems.

Concerning chromatin in non-mitotic plant cells, both the disturbance of protein phosphatase activities by MCYs and protein synthesis by CYN are likely to interfere with a wide range of intracellular events, leading to cell death. Programmed cell death (PCD) of plant cells bears several particularities, like: (i) It is not always accompanied by internucleosomal DNA cleavage (DNA laddering) (see [34], for an example); (ii) Due to the presence of cell wall, phagocytosis of apoptotic cells by neighbouring cells is not possible [35] and (iii) Subsequent PCD and necrosis in plant cells are more common, than in animal cells [36]. Cyanotoxin effects on plant PCD will be discussed in Section 5.2 and Section 6.2. 

## 4. An Overview of the Effects of MCY and CYN in Plants

There are a significant number of papers dealing with the effects of cyanotoxins on plants (see reviews [15,17,37,38,39,40] for examples). These include alterations of growth, development, anatomy, cell structure and physiological/biochemical processes as summarized on Table 1. Many of the effects listed can be directly related to the PP1 and PP2A inhibitory effect of MCYs and protein synthesis inhibitory effect of CYN. Other changes may be non-specific that is, not related directly to the biochemical effects of cyanotoxins (see Table 1 and later sections). All those alterations raised the need for the study of plant cellular changes that could reveal fine mechanisms of toxin action. Furthermore, growth and morphological changes did help in the understanding of cellular effects studied later. For example growth inhibition and histological marks of cell death can be related to the induction of PCD and non-specific cell death (necrosis) in plant cells. The cytological effects—focusing on chromatin and cytoskeletal changes will be discussed in the context of all levels (from organism to enzymes) of cyanotoxin effects in the following sections.

**Table 1:** Microcystin and cylindrospermopsin induced alterations in some vascular plants.

Cyanotoxins used in the experiments	Type of the cyanotoxin induced alterations	Plant taxon	References
I. Growth alterations			
MCY-LR, -RR, -LF, -FR, -LW, -YR in purified, -LR, -RR, -FR, -YR in extract, *M. aeruginosa* bloom samples	Inhibited seed germination, growth/elongation of shoot, primary root, leaves, inhibited increase of frond number, fresh/dry weight	*Brassica napus*, *Ceratophyllum demersum*, *Lemna minor*, *L. gibba*, *L. japonica*, *Lens esculenta*, *Lepidium sativum*, *Lolium perenne*, *Malus pumila*, *Medicago sativa*, *Myriophyllum variifolium*, *Oryza sativa*, *Phragmites australis*, *Pisum sativum*, *Sinapis alba*, *Spirodela oligorrhiza*, *Triticum durum*, *Vallisneria natans*,* Vicia faba*,* Vicia faba* inoculated with rhizobial strains,* Wolffia arrhiza*,* Zea mays*	[38,41,42,43,44,45,46,47,48,49,50,51,52,53,54,55,56,57,58,59,60,61,62,63,64,65,66,67,68,69]
MCY-RR	Decreased cell viability	Tobacco BY-2 cells	[70]
CYN (purified, crude extract)	Inhibited seed germination, growth/elongation of whole plant, shoot and mainroot, inhibited increase of fresh weight of leaves	*Brassica oleracea var. sabellica*,* Brassica juncea*,* Lactuca sativa*,* Lemna minor*, callus-derived *Phragmites australis* plantlets,* Sinapis alba*, *Wolffia arrhiza*	[52,71,72,73,74,75]
CYN in crude extracts	Concentration- and exposure time/exposed organ dependent stimulation or inhibition of growth	*Hydrilla verticillata*,* Lactuca sativa*,* Oryza sativa*, *Phaseolus vulgaris*,* Pisum sativum*,* Solanum lycopersicum*,* Spirodela oligorrhiza*	[74,76,77,78]
CYN	Inhibited pollen germination	*Nicotiana tabacum*	[79]
II. Morphological/Developmental alterations			
MCY-LR, -RR purified and in crude extract	Inhibited root elongation and altered primary root/lateral root formation, missing root hairs/crown root formation, radial expansion in roots, root coalescence	*Lens esculenta*, *Oryza sativa*, *Phaseolus vulgaris*, *Phragmites australis*, *Pisum sativum*, *Sinapis alba*, *Triticum durum*, *Vallisneria natans*, *Zea mays*	[43,46,51,52,56,58,60,69,80,81]
MCY-LR, -RR, -YR purified and in crude extract	Inhibited photomorphogenesis of cotyledons: Chlorotic and smaller cotyledons, missing trichomes of petioles, malformed, chlorotic fronds/leaves, inhibited shoot elongation, the seedlings lying horizontally on the paper bed. Stimulation of flowering	*Brassica napus*, *Lemna minor*, *Sinapis alba*, *Spinacia oleracea variants*	[45,51,52,53,82]
MCY-LR	Inhibited shoot and root formation, decrease of somatic embryo number	*Phragmites australis*, *Solanum tuberosum* tissue cultures	[46,58]
CYN	Increased root number, inhibited elongation, radial expansion of roots	*Hydrilla verticillata*, callus-derived *Phragmites australis* plantlets, *Sinapis alba*	[69,72,77]
CYN	Inhibited photomorphogenesis of cotyledons: The chlorotic, smaller cotyledons were violet colored in consequence of high level of anthocyanins	*Sinapis alba* seedlings	[52]
CYN in crude extract	Prolonged (9 days) exposure induced decrease of water content, browning, green color lost, shrunk leaves	*Oryza sativa*	[78]
III. Histological and cytological alterations			
MCY-LR	Lignification in cell walls (in cortical parenchyma, endodermis and pericycle, with increased autofluorescence)	*Phragmites australis* plantlets,* Sinapis alba* seedlings	Section 5.2; [58,69,80]
MCY-LR	Swelling of cells and formation of a callus-like tissue (in main roots and at the transit between main root and hypocotyls in mustard, in rhizome and roots of reed)	*Phragmites australis* plantlets,* Sinapis alba* seedlings	Section 5.2; [58,69,80]
MCY-LR	Early aerenchyma formation	*Phragmites australis* plantlets	[58]
MCY-LR	Inhibition of formation of vascular cylinder, xylem differentiation (xylem area and number of vessel elements)	*Phaseolus vulgaris*, *Sinapis alba* seedlings	Section 5.2; [60,69]
MCY-LR, -RR, -YR purified and in crude extract	Cell death by necrosis in cotyledon, shoot and root tissues	*Brassica napus*, *Lemna minor*, *Phaseolus vulgaris* seedlings, *Phragmites australis*, *Solanum tuberosum* tissue culture, *Sinapis alba* seedlings	Section 5.2; [43,46,51,52,53,55,58,69,80,83]
CYN	Lignification in cell walls was detected in some endodermis and pericycle cells at high CYN concentration	*Sinapis alba* seedlings	[69]
CYN	Formation of callus-like tissue and necrosis in reed root cortex, cell swelling in pith tissue without necrosis in mustard	*Phragmites australis* plantlets, *Sinapis alba* seedlings	[69,72]
CYN	Inhibition of xylem differentiation	*Sinapis alba* seedlings	[69]
IV. Physiology			
MCY-LR purified and MCY-RR, -LR, -YR, -(H4)YR, -WR, and -FR in crude extract	Inhibition/alteration of photosynthesis, decreased chlorophyll, carotenoid content, altered chl *a*/chl *b* ratio, alterations in chlorophyll fluorescence parameters	*Ceratophyllum demersum*, *Elodea canadensis*,* Lemna minor*, *L. gibba*,* Lens esculenta*,* Lolium perenne*, *Myriophyllum spicatum*,* Phaseolus vulgaris*, *Phragmites australis*,* Pisum sativum*, *Potamogeton sps.*, *Sinapis alba*,* Solanum tuberosum* tissue culture, *Spinacia oleracea* variants, *Spirodela oligorrhiza*, *Triticum durum*,* Vicia faba* inoculated with rhizobial strains, *Zea mays*	[45,46,47,48,52,59,61,62,63,64,68,82,83,84,85]
MCY-LR	After transient induction, inhibited anthocyanin accumulation in the cotyledons	*Sinapis alba*	[51,52]
MCY-LR, *M. aeruginosa* toxic culture	Inhibition of medium, nutrient uptake/absorbtion rates of phosphorus and nitrogen, nitrogen assimilation; Increase of mineral nutrients content in roots per fresh weight	*Lens esculenta*, *Phaseolus vulgaris*, *Pisum sativum*, *Potamogeton* sps., *Triticum durum*, *Vicia faba* inoculated withrhizobial strains, *Zea mays*	[46,61,67,68,85]
MCY-LR	Decreased water and protein content	*Ceratophyllum demersum*	[63]
CYN in crude extract	Decreased chlorophyll content or/and changes in the chl- *a*/chl-*b* ratio	*Hydrilla verticillata*, *Sinapis alba*	[52,77]
CYN purified and in crude extract	Soluble protein content per unit fresh weight showed mild increases, especially in *W. arrhiza*, increases of tubulin content in reed roots	*Lemna minor*, *Phragmites australis* plantlets, *Wolffia arrhiza*	[72,73]
V. Enzymology			
MCY-LR, MCY-RR, -LW, -LR, -LR in crude extract	Inhibition of protein-phosphatases PP1 and PP2A: * in vitro* inhibition of active forms of PP1 and PP2A in diluted seed extract (both PP1 and PP2A IC_50_: ~0.1 nM); *in vivo* inhibition of PP1 and PP2A	*Brassica napus* seed extract, *Medicago sativa*, *Phragmites australis*, *Sinapis alba*	[9,43,69,80,86]
MCY-LR	Inhibition of PP1 and PP2A, blocking of sucrose-inducible gene expression (mRNAs of β-amylase, sporamin, AGPase)	*Ipomoea batatas*, transgenic* Nicotiana tabacum*	[87]
MCY-LR	Inhibition of PP2A, the major sucrose-phosphate synthase (SPS) phosphatase blocking of the light-induced activation of SPS and decreasing sucrose biosynthesis and CO_2_ fixation	*Spinacia oleracea*	[88]
MCY-LR	Disturbance of jasmonic acid (JA) signal transduction; abrogation of the response to JA (both the increase in the specific activity of acid phosphatase (AP) and the reduction in overall protein content shows opposite tendency)	*Nicotiana tabacum*	[89]
MCY-LR (purified and in extract)	Alterations in activities of hydrolase enzymes: Changes in activity of constitutive acid phosphatase and RNase; induction of ssDNase activities; PCD associated changes of ssDNase and dsDNase activities in plant cells	*Spirodela oligorrhiza* *Sinapis alba* *Phragmites australis*	[48,51,90]
MCY-LR, -LF, -LR in extract	Lipid peroxidation, increased α- and β-tocopherol concentration (as a lipid antioxidant)	*Arabidopsis thaliana* cell suspension,* Medicago sativa*, *Triticum aestivum*	[91,92,93]
MCY-LR, -RR, -LF, -LW, -WR both in purified and in crude cyanobacterial extracts	Phenomena induced by oxidative stress: *in vitro* reaction of MCY-LR and extracted plant GST producing GSH-MCY-LR conjugate, identification of* in vivo* formed GSH-MCY-LR conjugate; formation of H_2_O_2_, other ROS, increase in phenolic compounds, phenylalanine ammonia lyase (PAL), polyphenol oxidase (PPO) activities, concentration of endogenous nitric oxide (NO); decrease/alterations in glutathione pool; reduced glutathione (GSH) and glutathione disulfide concentration; induction/alterations of oxidative stress enzyme activities: microsomal and cytosolic/soluble glutathione-*S*-transferase (mGST and sGST), -peroxidases (GPx), -glutathione reductase (GR), ascorbate peroxidase (APX, POD), superoxide dismutase (SOD), catalase (CAT)	*Arabidopsis thaliana* cell suspension, *Brassica napus*,* Brassica rapa*, *Ceratophyllum demersum*, *Elodea canadensis*, *Lemna gibba*, *L. minor*, *Lepidium sativum*, *Medicago sativa*, *Myriophyllum spicatum*, *Oryza sativa*, *Phaseolus vulgaris*, *Phragmites australis* , *Sinapis alba*, *Spinacia oleracea* variants, tobacco BY-2 cell suspension, *Triticum aestivum*, *Vicia faba* inoculated with rhizobial strains, *Vigna unguiculata* species variants	[38,50,52,53,55,59,62,65,68,70,82,91,92,94,95,96,97,98,99,100]
MCY-LR in extract	Inhibited production of nitric oxide (NO), decreased auxin (IAA) concentration in roots	*Oryza sativa*	[81,101]
CYN	Alteration in protein synthesis: CYN inhibited the eukaryotic protein synthesis apparatus with similar potency in plant and mammalian cell extracts, partial inhibition of protein production in germinating pollen tubes	wheat germ extract, *Nicotiana tabacum*	[79,102]
CYN	Significantly decreased PP1 and PP2A activities in extracts of CYN treated plants (CYN did not cause significant decrease in PP1 activity * in vitro*)	*Sinapis alba*	[69]
CYN purified and in crude extract	Protease isoenzyme activity gels showed significant alterations in protease enzyme pattern and activities; crude extract induced an increase of total protease activity at pH 5 and pH 8, while purified CYN increased the activity only at lower concentration regimes (0.01—1 μg mL^−1^)	*Lemna minor*, *Wolffia arrhiza*	[73]
CYN purified and in crude extract	Induction of oxidative stress enzyme activities: Increased GST, GPx activities; increased POD activity only at low (0.05 μg mL^−1^) concentration—transient effect	*Oryza sativa*, *Sinapis alba*	[52,78]

## 5. Effects of MCY on Plant Mitotic and Non-Mitotic Chromatin

### 5.1. Mitotic Chromatin

MCY-LR exerts a characteristic dose-dependent effect on the mitosis of animal cells. It stimulates transiently mitotic activity (at low doses of the toxin or at shorter exposure times). In contrast, at higher MCY-LR doses, mitosis is inhibited, that is correlated with the induction of PCD and necrosis [103,104]. This phenomenon was named as “dualistic response” and it is probably related to the following mechanism: At low concentrations, the partial inhibition of PP1 and PP2A induces the maintenance of phosphorylation state of certain mitogen activated protein kinases (MAPKs), thereby activates them, causing stimulation of entry to the M phase of cell cycle. A severe inhibition of PP1 and PP2A will induce a general disturbance of cell functioning that finally leads to cell death [104].

Similar observations have been made in plants. Stimulation of mitosis was observed at low MCY-LR concentrations in several model systems, while inhibition of mitotic activity occurs in long-term treated, non-synchronized root tip meristematic cells of *Phragmites australis* and *Sinapis alba*. The inhibition of mitosis could be correlated to the inhibition of growth. At a close examination of distinct mitotic phases, the toxin stimulates early as well as late mitosis at different doses at relatively long-term exposures of roots [58,69,80,105]. A characteristic effect could be observed in shoot tip meristems of *Ceratophyllum demersum*, where MCY-LR clearly blocked cells in prophase/prometaphase [63]. Further studies were focused on root meristems in order to elucidate the mechanisms of MCY-LR induced mitotic changes. In non-synchronized root cells treated at relatively long term with MCY-LR a total arrest cells in early mitosis was not observed, but at certain concentrations it increased early mitosis indices, indicating that it may either inhibit progression of mitosis or change the duration and inhibit exit from mitosis [80,105]. Interestingly, dose-dependent short-term (4–30 h) toxin exposures of hydroxylurea-synchronized cells of *V. faba* showed apparently opposite effects: At low doses (1 μg mL^−1^), MCY-LR did not increase significantly the rate of cell division, but increased late mitosis index and delayed metaphase-anaphase transition [105]. This effect on early-late mitosis transition was similar to that observed in *Tradescantia* stamen hair cells microinjected with the toxin [106]. Since disturbances in the onset of anaphase were observed in MCY-LR treated mammalian (CHO-K1) cells too [103], this effect of the toxin might be universal in eukaryotic cells. At higher dose (10 μg mL^−1^), there was a delayed entry into M phase, but the acceleration of cell cycle and overall stimulation of mitosis, with a relatively low rate of late mitosis in *V. faba* [105]. These effects show that depending on the doses used at short-term treatments, MCY-LR delays entry to mitosis or the transition of cells to late mitosis and exit from late mitosis but allows the onset of a new cell cycle. Thus, it does not block completely progression of mitosis. What could be the cause of this? Since the toxin exerted its effects at low exposure times, one may assume that those effects could be correlated directly to the inhibition of PP1 and PP2A and the consequent hyperphosphorylation of proteins involved in the regulation of late mitosis. In addition, protein phosphatase inhibition assay showed that mitotic alterations are correlated with decreases of PP1 and PP2A activities in *P. australis* roots exposed to MCY-LR for 10 days [80]. Thus, the functioning of specific proteins could be changed. Indeed to date, three phenomena could be detected in plant cells (i) alterations in the organization of mitotic MTs; (ii) histone H3 hyperphosphorylation; (iii) abnormal sister chromatid segregation, the appearance of lagging chromosomes and micronuclei at the end of mitosis. Below, we will analyze these phenomena in detail.

**Figure 1:**
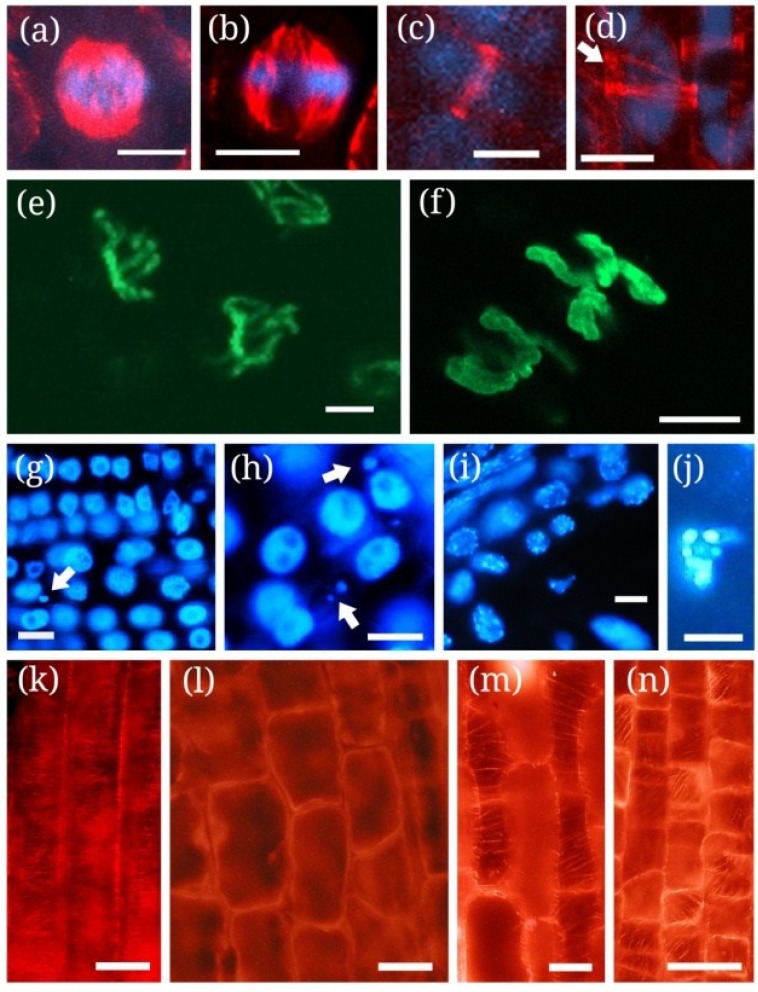
Conventional fluorescence (**g**–**n**) and confocal microscopy (**a**–**f**) images of characteristic chromatin and microtubule alterations induced by cyanotoxins in plant cells as revealed by histochemical and immunohistochemical methods (see [105] as an example for methods). Chromatin label is shown in blue, Ser10-phosphorylated histone H3 in green and microtubules in red. (**a**) Normal metaphase spindle from* in vitro* cultured control *Phragmites australis* root tip meristem; (**b**) *P. australis* root tip meristem cell treated with 10 μg mL^−1^ MCY-LR. Note abnormal bundling of microtubules and spindle disruption; (**c**) Prophase cell of a *P. australis* root tip with normal preprophase band (PPB); (**d**) Split PPB (arrow) of a *P. australis* cell treated with 10 μg mL^−1^ CYN; (**e**) Control *Vicia faba* root tip meristem cell labeled for phospho-histone H3 Ser10. Histone H3 is phosphorylated mainly at the pericentromeric regions of metaphase chromosomes; (**f**) *V. faba* cell treated with 20 μg mL^−1^ MCY-LR. Note histone H3 hyperphosphorylation both at pericentromeric regions and chromosome arms; (**g**) Nuclei of *V. faba* meristematic cells labeled with DAPI. Micronuclei occur only sporadically (arrow); (**h**) Abundance of micronuclei in *V. faba* meristem treated with 20 μg mL^−1^ MCY-LR (arrows); (**i**) Nuclei of *Sinapis alba* cells from root elongation zone labeled with DAPI. No micronuclei or nucleus fragmentation can be detected; (**j**) Fragmented nucleus of a *Sinapis alba* cell from root elongation zone treated with 1 μg mL^−1^ MCY-LR; (**k**) Control *P. australis* cells from root elongation zone, with normally oriented cortical microtubules (CMTs); (**l**) *P.australis* root cells treated with 20 µg mL^−1^ MCY-LR showing CMT depolymerization and inhibition of cell elongation; (**m**,**n**) *P.australis* root cells treated with 10 μg mL^−1^ CYN, showing a cell with decrease of MT density (**m**) and a cell with CMT reorientation, inhibition of elongation and stimulation of radial expansion of cells at the transition of meristematic-elongation zone (**n**). Scalebars: 15 μm (**a**,**b**), 10 µm (**c**,**d**,**g**–**j**), 5 µm (**e**,**f**); 30 µm (**k**,**l**), 25 µm (**m**,**n**). Micrographs taken by D. Beyer (**a**,**b**,**e**,**f**), C. Máthé (**g**–**n**) and J. Roszik (**c**,**d**).

Several studies proved MCY-induced alterations of mitotic spindle formation and consequently, abnormal chromosome movement during animal and human cell division. Characteristic anomalies were monopolar, multipolar or disrupted spindles [103,107,108,109]. Similar spindle anomalies were observed in plant cells, too ([69,80,105]; Figure 1a,b of this study). These alterations are important in the context of chromatin dynamics during mitosis: all of them are correlated with abnormal sister chromatid segregation [103,110,111]. This was the situation in MCY-LR treated plant cells ([69,80,105]; Table 2 of this study). Since plant cells possess diffuse MTOCs and not centrioles, these similarities may indicate that besides MT nucleation, other regulatory mechanisms common to animal and plant cells may occur at MCY exposures. Moreover, the disturbance of phragmoplast organization was observed in MCY-LR treated cells that may be due to mechanisms similar to the formation of spindle anomalies [80,105]. MAPs are good candidates to such mechanisms: Their binding to MTs is involved in the regulation of correct MT assembly/disassembly, bundling and stability both in animal and plant cells [26,33,108]. The binding of several MAPs is of crucial importance for correct mitotic MT assembly and depends on their phosphorylation state. For example, proteins of the MAP65 family found in *Arabidopsis thaliana* and *Nicotiana tabacum* are not able of mitotic or non-mitotic MT binding, when they are phosphorylated, e.g., PP1 and PP2A do not dephosphorylate them [32,112]. AtMAP65-1 regulates mitotic spindle and phragmoplast assembly by promoting MT bundling. Its hyperphosphorylation leads to spindle destabilization and blocks phragmoplast expansion [32]. Therefore, the same post-translational modification of a MAP alters two types of mitotic MT organization. It should be noted that the Arabidopsis *ple4* genotype that bears a mutation in the *AtMAP65-3/PLEIADE* gene is characterized by alteration of AtMAP65-1 binding to MTs and multinucleate cells in root meristems: this mutant shows a clear relationship between mitotic MT assembly and correct chromatid segregation [113]. We can conclude that phosphorylation dependent MAPs might be targets of MCY action in plant cells and could be related to the direct biochemical effects of the toxin (*i.e*., PP1 and PP2A inhibition) on mitotic and non-mitotic MT dynamics and consequent chromatin organization. However, such studies are still awaited. 

**Table 2:** A survey of the effects of MCY and CYN on plant chromatin organization.

Cyanotoxin	Plant material	Effect	Mechanisms elucidated or probably involved	References
1.Mitotic chromatin				
MCY-LR, MCY-RR	*Tradescantia virginiana* stamen hair cells	Increase of metaphase transit time, temporary delay of sister chromatid segregation	Inhibition of serine-threonine protein phosphatases (type 1 and 2A)	[106]
MCY-LR	Root tip meristems of *Phragmites australis*, *Sinapis alba*	Dualistic response: Mitotic activity increases at low, decreases at high cyanotoxin concentrations	Inhibition of serine-threonine protein phosphatases (type 1 and 2A)	[69,80]
MCY-LR	Root tip meristems of *P. australis*, *Vicia faba*	Transient increase of early and late mitotic activity	Inhibition of serine-threonine protein phosphatases (type 1 and 2A)	[80,105]
MCY-LR	Shoot tip meristems of *Ceratophyllum demersum*	Arrest of mitosis in prophase/prometaphase	Blocking of MT dynamics at early mitosis; inhibition of serine-threonine protein phosphatases (type 1 and 2A)	[63]
MCY-LR	Root tip meristems of* V. faba*	Acceleration of cell cycle at exposure to high (10 μg mL^−1^) toxin concentration	Inhibition of serine- threonine protein phosphatases (type 1 and 2A)	[105]
MCY-LR, MCY-XR	Root tip meristems of* P. australis*, *S. alba*, *V. faba*, *Allium cepa*	Delay of metaphase/anaphase transition, incomplete sister chromatid segregation, the formation of micronuclei	Disruption of mitotic MT structures; inhibition of serine-threonine protein phosphatases (type 1 and 2A); * Hyperphosphorylation of histone H3 at Ser10	[69,80,105,114]
CYN	Root tip meristems of* P. australis*	Alteration of early mitotic activity (increase of prophase/prometaphase, decrease of metaphase indices)	* Alteration of PPB development, probably due to protein synthesis inhibition	[72]
CYN	Root tip meristems of* P. australis*, *S. alba*	Alteration of sister chromatid segregation	Disruption of mitotic MT structures	[72]
2. Non-mitotic chromatin				
MCY-RR	Tobacco BY-2 cells	Perinuclear chromatin marginalization	Oxidative stress (ROS generation)	[115,116]
MCY-LR, MCY-RR	Tobacco BY-2 cells, *Vallisneria natans* mesophyll cells, *P. australis* root tips	Chromatin condensation	Oxidative stress, induction of SSP nuclease activities	[90,115,117,118]
MCY-RR, MCY-LR	Tobacco BY-2 cells, *S. alba* roots	Nuclear fragmentation	Oxidative stress	Figure 1j and Figure 2g-j, [115]
MCYs (cyanobacterial extract)	*Oryza sativa* seedlings	DNA fragmentation (smearing)	Probably oxidative stress	[119]

* Specific effect, probably due to the direct biochemical action of the given cyanotoxin.

An early proof showing that MCY-LR interferes with histone H3 phosphorylation at Ser10, came from the study of maize meiocytes [120]. Later on it turned out that in relation to mitotic chromatin dynamics, one of the most important post-translational modifications of histone H3 and its counterpart, centromeric histone H3 (CENH3, CENPA) is phosphorylation at specific Ser/Thr residues at their N-terminal regions [121,122]. These types of histone modifications are detected most commonly in mitotic and meiotic plant cells: during mitosis, phosphorylation starts in prophase, culminates in pericentromeric and centromeric regions of metaphase chromosomes and decreases gradually at late mitosis to be practically undetectable at the end of cytokinesis [121,122]. They are important for correct chromatin condensation and cohesion at prophase-metaphase and sister chromatid segregation at anaphase in all eukaryotic cells [123,124]. As an inhibitor of PP1 and PP2A, MCY-LR was expected to induce histone H3 hyperphosphorylation during plant mitosis. Indeed, in *V. faba* root tips the toxin induced high levels of phosphorylation not only at the pericentromeric regions, but in chromosome arms as well. This was accompanied by chromosome hypercondensation in metaphase and the formation of lagging chromosomes leading to the formation of micronuclei (Figure 1e–h; [105]). Therefore, sister chromatid segregation anomalies induced by MCY are related not only to the formation of aberrant mitotic MT structures, but on direct modifications at the chromatin level, too. Histone H3 hyperphosphorylation could be a suitable marker for the effects of MCY at the chromatin level. Whether the toxin induces this histone modification directly through the inhibition of PP1 and PP2A, or the modification is the result of hyperphosphorylation of histone-regulatory proteins active during the M phase of cell cycle, should be subject of future research. For example, the hyperphosphorylation of certain Aurora kinases induces their activation, that is, their capacity of histone phosphorylation [125]. Therefore one may assume that MCY-LR, as a phosphatase inhibitor induces histone H3 hyperphosphorylation not only by the direct inhibition of its dephosphorylation, but through hyperactivation of such kinases as well in *V. faba*. It should be noted however, that Aurora kinases with important roles in H3 phosphorylation are localized in centromeric regions of plant chromosomes [126,127]. Since we observed that MCY-LR induces H3 hyperphosphorylation both in pericentromeric regions and chromosome arms, it is unlikely that the toxin acts solely through this mechanism in *V. faba*: direct inhibition of H3 dephosphorylation may be more important in this respect. 

Microfilaments—the other component of plant cell cytoskeleton-play an important role in cell division and the determination of cell shape/movements in eukaryotic cells [27,128]. The alteration of protein dephosphorylation by other drugs than MCY induces alterations in F-actin organization in guard cells and soybean cultured cells [129,130]. MCY-LR does induce MF disruptions in animal cells and this was related to programmed cell death or necrosis [107,109,131]. To our best knowledge, similar studies on plant cells are lacking, so this issue needs to be studied.

### 5.2. Non-Mitotic Chromatin

It has been known for a long time that MCY induces PCD/apoptosis or necrosis in animal cells (see [109,132] for examples). In an early study, nuclear fragmentation induced by MCY-LR was detected in different rat cell types [133]. It should be noted that such nuclear fragmentation/micronucleus formation could not be detected in MCY-LR treated young mouse erythrocytes or human lymphocytes [134].

**Figure 2:**
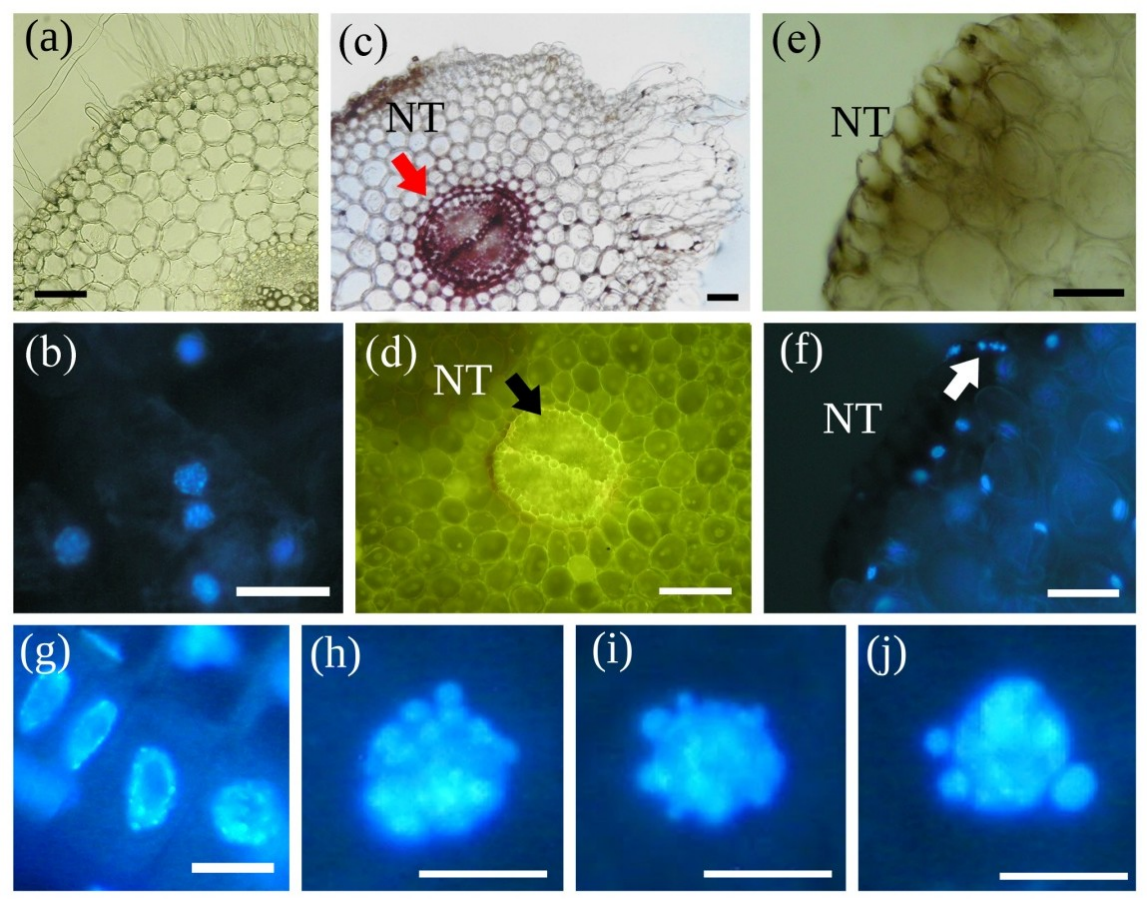
MCY-LR induces callus formation (swollen cells), PCD and necrosis in the rhizodermis and cortex of *S. alba* primary roots. (**a**) Control root collar; (**b**) Same tissues stained with DAPI; (**c**) Cross-Section of main root-hypocotyl transition zone of seedlings treated with 20 μg mL^−1^ MCY-LR, showing the formation of a callus-like tissue (CA), necrosis (NT) and intensive lignification of endodermis and stele as shown by phloroglucin-HCl staining (arrow); (**d**) High autofluorescence of inner root tissues (arrow) induced by 20 μg mL^−1^ MCY-LR exposure: autofluorescence fades away in necrotic tissue (NT); (**e**) 20 μg mL^−1^ MCY-LR induces necrosis of rhizodermis and adjacent tissues (NT); (**f**) Same tissues as in (**e**) stained with DAPI, nuclei are absent in necrotic cells (NT), and the fragmentation of nuclei can be observed in cells neighboring necrotic tissue (arrow); (**g**–**j**) Nuclei from the root tip meristem-elongation zone transition; (**g**) control; (**h**–**j**) Treatment with 5 μg mL^−1^ MCY-LR: nuclear blebbing (**h**,**i**) leading to the fragmentation of chromatin (**j**); Scalebars: 80 μm (**a**,**b**,**e**,**f**), 200 μm (**c**,**d**) and 15 μm (**g**–**j**). Micrographs taken by M. M-Hamvas (**a**,**c**,**e**), C. Máthé (**b**,**d**,**f**–**j**).

MCY-LR induces the formation of micronuclei not only by the formation of lagging chromosomes, but in non-mitotic plant cells as well. Altered sister chromatid segregation is characterized by the formation of one micronucleus per cell (Figure 1g,h of this study). In non-dividing cells, the formation of multiple micronuclei and chromatin fragmentation could be observed in several studies ([115]; Figure 1i,j and Figure 2g–j of this study). This phenomenon is generally attributed to PCD [135,136] and shows the possible role of MCY in its induction in plants. Indeed, there is a significant number of studies showing the subsequent events leading to nuclear fragmentation in MCY treated plant cells. In general, firstly chromatin condensation and perinuclear chromatin marginalization occurs, followed by nuclear and finally, DNA fragmentation [90,115,116,117,118,137]. MCY induced internucleosomal DNA fragmentation (DNA laddering) has been observed in several rat and human cell types [137,138]. In contrast, this could not be detected in MCY treated plant cells undergoing PCD. Instead, a non-specific DNA fragmentation (smearing) was observed on agarose gels [119]. Therefore, MCY induced PCD or apoptosis, a type of PCD might develop by partially different mechanisms in plant and animal cells.

As we mentioned before Section 4, MCYs are able of inducing necrosis in a significant number of plant species/systems. Necrosis is a “non-specific”/not programmed cell death type characterized by general disruption of cell structure in eukaryotes and in particular, plant cells [118]. PCD/apoptosis and necrosis can be induced by different MCY-RR concentrations in tobacco BY-2 cells [117] and a similar finding has been made by Jámbrik* et al.* [90] in MCY-LR treated *P. australis* roots. However, the clear separation of apoptotic and necrotic effects of microcystins needs further study: for MCY-LR treated mustard roots, it seems that the same cell undergoes PCD, followed by necrosis: nuclear fragmentation occurs firstly and subsequent disappearance of DNA from cells is observed (Figure 2b,f). In relation to this, it is known that PCD/apoptosis and necrosis are frequently subsequent events in plants [36]. Although in several cases chromatin marginalization was thought to be essential for the initial steps of PCD, when *P. australis* roots were treated with high MCY-LR doses, chromatin marginalization could be associated to necrosis and not PCD [90]. Indeed, this type of chromatin change can be associated to initial steps of necrosis of animal cells [139]. 

What are the possible mechanisms of cell death induction by MCY in plants? Several studies in animal and plant cells indicate that MCYs induce the elevation of ROS and the activities of enzymes involved in oxygen radical scavenging ([52,70,91,98,99,140]; Table 1 of this study). MCYs are able of binding GSH, thereby of forming glutathione conjugates by enzymatic or non-enzymatic mechanisms in a variety of organisms including plants, invertebrates and fish [47,94]. This is a detoxifying mechanism, but it induces the reduction of GSH and general glutathione pool in cells, contributing to oxidative stress [94,98]. As a general mechanism, the elevation of ROS induces DNA damage. If repair mechanisms fail, increases in the activities of nucleases and proteases will lead either to chromatin condensation and fragmentation and finally, PCD, or to necrosis [141,142]. It is believed that MCYs induce such processes. ROS elevation was linked to DNA damage and/or nuclear degradation in animal (see [143] for an example) and in plant cells [115,116]. The cyanotoxin induced increase of protease and nuclease activities as well as increases in peroxidase activities involved in oxygen radical scavenging have been demonstrated in several terrestrial and aquatic model plant systems [51,52,73,90].

An alternative mechanism to MCY induced ROS elevation is related to the phosphatase inhibitory effect of cyanotoxin. According to this, the inhibition of protein phosphatases will influence signal transduction pathways that alter stress related gene expression, leading to decrease of reducing power and increase of ROS [144]. Although protein phosphatase inhibition in general can induce directly ROS elevation [145], there is not enough evidence for such particular effect of MCY in plants. It is worth mentioning that in animal cells, MCY-LR can induce the inhibition of dephosphorylation of proteins involved in ROS formation [146].

In addition to chromatin disorganization, PCD and necrosis is associated with MT destabilization [147]. Indeed, MCY-LR does induce CMT depolymerization (*Phragmites australis* root tips) or reorientation (*Ceratophyllum demersum* shoot tips). Besides cell death, these processes are correlated to changes of cell shape and alterations in the morphology of axial organs (Figure 1k,l; [63,80]). 

## 6. Effects of CYN on Plant Mitotic and Non-Mitotic Chromatin

### 6.1. Mitotic Chromatin

In animal cells, CYN induces a general inhibition of mitotic activity [148]. Moreover, it induces micronucleus formation via chromosome loss in human WIL2-NS lymphoblastoid cell line [22]. Micronucleus formation was observed both in dividing and non-dividing human Caco-2 and HepaRG cell lines, too [149]. Concerning plants, CYN induces transient increase of mitotic activity of *P. australis* root tip meristematic cells, but this is not observable in *S. alba* [69,72]. Therefore, a “dualistic response” or mitotic stimulation cannot be generalized yet for plants, as for MCY (see Section 5.1). However as for MCY, CYN induces spindle disruptions in both plant systems and disorganization of phragmoplasts in *P.* *australis.* These changes are accompanied with disturbances in sister chromatid segregation during anaphase [69,72]. The mechanisms laying behind these phenomena are largely unknown, but two hypotheses can be drawn out: (i) The inhibition of MAP synthesis, as CYN is a protein synthesis inhibitor; (ii) Since CYN inhibits PP1 and PP2A in a non-specific way [69,150], it may influence the functioning of phosphorylation-dependent MAPs, as MCY.

CYN induces the formation of double or split PPBs at least in *P. australis* roots ([72]; Figure 1c,d of this study). It remains to be established whether CYN induced PPB alterations are general in plants. Alterations in PPB assembly can be attributed to the protein synthesis inhibitory effect of the cyanotoxin, since other inhibitors, like cycloheximide have similar effects on plant cells [30]. In addition, disturbances of auxin transport (disfunctions of auxin transport proteins) induce the formation of abnormal PPBs, leading to changes in cell division plane [28]. PPB is believed to regulate cell division plane and in consequence, plant morphogenesis [26,30]; therefore, anomalies in its development may contribute to CYN induced changes in plant growth and morphogenesis (see Table 1). The possible mechanism of altered PPB formation is thought to be disturbance of MAP formation [72]. 

### 6.2. Non-Mitotic Chromatin

There is a significant number of studies on animal and human cells indicating that CYN induces PCD [17]. These include nuclear fragmentation/micronucleus formation in non-mitotic cells [149,151] and DNA degradation as shown by electrophoresis of purified DNA and the Comet assay [142,151,152]. The elevation of ROS and its possible role in the development of DNA damage and PCD/apoptosis has also been shown in fish as well as human cell lines [153,154]. However a recent study has shown that although CYN induces ROS elevation, it is not correlated with DNA damage and PCD in HepG2 cells [155]. Therefore, the correlation of CYN induced ROS elevation and DNA degradation is still controversial for animal and human systems. In animal cells (CHO K1 cells), CYN induces cytoskeletal reorganization, thereby inhibiting growth and inducing PCD and necrosis [109,156]. 

To date, there are no published data on the effects of CYN on chromatin organization in differentiating plant cells. However, several studies indicate that the cyanotoxin may induce PCD in plant cells. These include: (i) Increases in several protease isoenzyme activities in the aquatic plants *Lemna minor* and *Wolffia arrhiza* [73]; (ii) Increasing of enzyme activities that indicate the elevation of ROS and oxidative stress: GST, glutathione peroxidase in rice roots, peroxidase in *Sinapis alba* seedlings [52,78]; (iii) CYN reduces MT density and induces MT reorientation in *Phragmites australis* root cells in the meristematic and elongation zone of roots that inhibit growth, alter root morphology and is probably related to cell death. Reduction of MT density is not caused by the inhibition of tubulin synthesis, since CYN increases the production of this protein. Rather, it can be correlated to the synthesis and functioning of MAPs involved in the regulation of cytoskeletal stability (Figure 1k,m,n; [72]).

It is worth mentioning that the cyanotoxin induces the formation of necrotic-like tissue in *P. australis* roots, but not in *S. alba* seedlings [69,72]. Other plant systems should be studied in this respect in order to reveal a general rule for higher plants concerning induction of cell death. 

## 7. Conclusions

**Figure 3:**
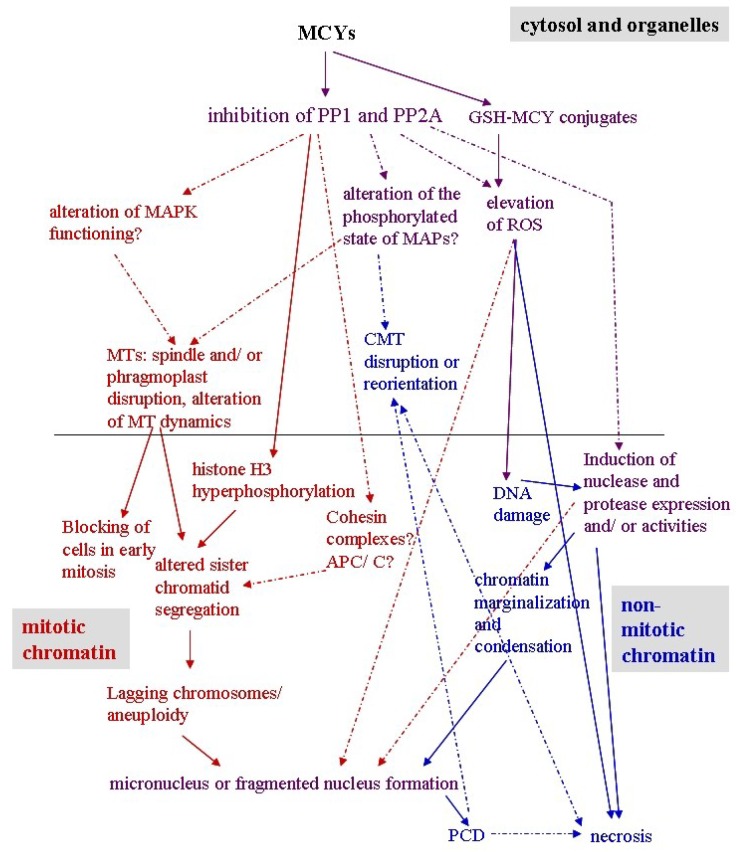
Proposed network for the effects of MCY on plant chromatin organization and functioning. Continuous arrows represent pathways already elucidated, while dashed arrows or question marks represent cellular processes or pathways that need further proof. Color code: red—mitotic cells, blue—non-mitotic cells, purple—all cell types. Abbreviations: APC/C-anaphase promoting complex-cyclosome; CMT-cortical microtubules; GSH-reduced glutathione; MAP-microtubule associated protein; MAPK-mitogen activated protein kinase; MT-microtubule; PCD-programmed cell death; PP1-protein phosphatase of type 1; PP2A-protein phosphatase of type 2A; ROS-reactive oxygen species.

The effects of MCY and CYN on chromatin organization in plants are summarized on Table 2 and Figure 3. MCY induces anomalies at the chromatin level in mitotic plant cells, namely it alters sister chromatid segregation leading to the formation of lagging chromosomes which will turn into micronuclei as they decondense at the end of mitosis. To date, two main mechanisms have been elucidated in relation to this: (i) Abnormal organization of mitotic MTs, including disruption of mitotic spindles and phragmoplasts, and the formation of monopolar/multipolar spindles; (ii) Hyperphosphorylation of histone H3. These processes can be correlated directly to the protein phosphatase inhibitory effect of the toxin. Concerning non-dividing cells, MCY induces changes characteristic for the onset of PCD and/or apoptosis: Chromatin marginalization and condensation, nuclear fragmentation, DNA degradation. This is probably related to the elevation of ROS and increases of SSP nuclease and protease activities.

Concerning CYN, it induces spindle-phragmoplast disruption and abnormal PPB formation, probably due to its protein synthesis inhibitory effects in mitotic plant cells. To date, there is no direct evidence of CYN induced PCD in plant cells, but there are several indirect proofs e.g., modulation of protease activities and increases in ROS and oxygen radical scavenging enzymes-that raise the possibility of inducing this phenomenon. CYN-induced necrosis has been proven in* in vitro* grown *P. australis* plantlets only.

A generalized diagram for the effects of MCY on plant chromatin is shown on Figure 3. It should be noted that concerning CYN, there is still little knowledge in plants concerning this topic.

As proposed in Section 5 and Section 6, the effects of cyanotoxins on plant chromatin organization raise many future questions and research directions. These will contribute to a better understanding of the effects of MCY and CYN on plant cells and will serve as tools for a general understanding of plant cell functioning.
